# Risk Preferences and Prenatal Exposure to Sex Hormones for Ladinos

**DOI:** 10.1371/journal.pone.0103332

**Published:** 2014-08-01

**Authors:** Diego Aycinena, Rimvydas Baltaduonis, Lucas Rentschler

**Affiliations:** 1 Centro Vernon Smith de Economía Experimental, Universidad Francisco Marroquín, Guatemala City, Guatemala; 2 Department of Economics, Gettysburg College, Gettysburg, Pennsylvania, United States of America; 3 Centro Vernon Smith de Economía Experimental, Universidad Francisco Marroquín, Guatemala City, Guatemala; University of Turku, Finland

## Abstract

Risk preferences drive much of human decision making including investment, career and health choices and many more. Thus, understanding the determinants of risk preferences refines our understanding of choice in a broad array of environments. We assess the relationship between risk preferences, prenatal exposure to sex hormones and gender for a sample of Ladinos, which is an ethnic group comprising 62.86% of the population of Guatemala. Prenatal exposure to sex hormones has organizational effects on brain development, and has been shown to partially explain risk preferences for Caucasians. We measure prenatal exposure to sex hormones using the ratio of the length of the index finger to the length of the ring finger (2D:4D), which is negatively (positively) correlated with prenatal exposure to testosterone (estrogen). We find that Ladino males are less risk averse than Ladino females, and that Ladino males have lower 2D:4D ratios than Ladino females on both hands. We find that the 2D:4D ratio does not explain risk preferences for Ladinos. This is true for both genders, and both hands. Our results highlight the importance of exploring the behavioral significance of 2D:4D in non-Caucasian racial groups.

## Introduction

Risk preferences play an important role in human decision making (i.e. investment choices, career and health choices, etc.). Understanding the underlying determinates of these preferences will thus refine our understanding of human behavior in a broad array of environments.

A robust finding from psychology is that men display more risk seeking behavior than women. In a meta-study of 150 studies, men were found to be less risk averse than women in 14 out of 16 risky tasks [1]. In a survey of studies, in which financially incentivized risk elicitation tasks are used, [2] show that men are more risk seeking than women in abstract gambles, although gender differences are less pronounced when the task is contextualized (for example, framed as an investment decision).

One possible explanation for these behavioral differences is that variations in the prenatal exposure to sex hormones are a driving factor. The exposure to sex hormones in utero has implications for organizational brain development [3]. A measure of such exposure is the length of the second finger relative to the length the fourth finger (2D:4D), which is negatively (positively) correlated with exposure to testosterone (estrogen) in utero [4]–[8]. This ratio is fixed early in development, and remains stable [5], [8], [9]. An advantage of using 2D:4D rather than circulating hormones, is that 2D:4D is unambiguously exogenous. Further, circulating hormones and the 2D:4D ratio are not correlated [10].

The 2D:4D ratio is sexually dimorphic. In particular, men have, on average, a lower 2D:4D ratio than women on both hands [8]. It is important to note that 2D:4D is also sensitive to race, so it is important to consider racially homogenous samples [11], [12].

The evidence regarding the relationship between 2D:4D and risk preferences is mixed. [13] finds that a gender difference in the 2D:4D ratio is able to partially explain the gender difference in risk preferences for a sample of Caucasians, and that the relationship between risk taking behavior and 2D:4D is nonlinear. [14] also finds a relationship between 2D:4D and risk preferences for men in a sample of Caucasians. However, [15]–[17] all find no effect when using racially heterogeneous samples.

We consider, for the first time, the relationship between risk preferences and 2D:4D for a racially homogenous non-Caucasian sample. We analyze a sample of Guatemalan Ladinos. Ladino is an ethnic group which comprises 62.86% of the population of Guatemala, and 92.55% of 18–26 year olds with a college education in the Guatemala City metropolitan area [18]. In addition, this paper is the first systematic analysis of 2D:4D for a Ladino population. We investigate the hypothesis that there is a (possibly nonlinear) relationship between risk preferences and 2D:4D for Ladinos. We also investigate differences in 2D:4D between Ladinos and Caucasians.

## Results and Discussion

### Summary statistics and gender

We first consider the elicited risk preferences of both males and females. As mentioned above, risk preferences are elicited using an incentivized task similar to that of [19]. See the Methods section for a detailed description of this procedure. Our measure of risk aversion is the number of safe choices a participant makes. Rather than imposing consistency on the expressed preferences, we opted to allow participants to make inconsistent choices (which may be driven by indifference between the lotteries, or confusion) so that we can account for this in our analysis. In [13] risk elicitation tasks similar to those from [20] are used, which do not allow for inconsistent choices. We find that the proportion of participants who exhibit inconsistent choices in the risk elicitation task does not differ across gender (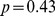). In our analysis of risk preferences, we thus restrict attention to those participants who made consistent choices. All results are robust to using the full sample.

**Figure 1:**
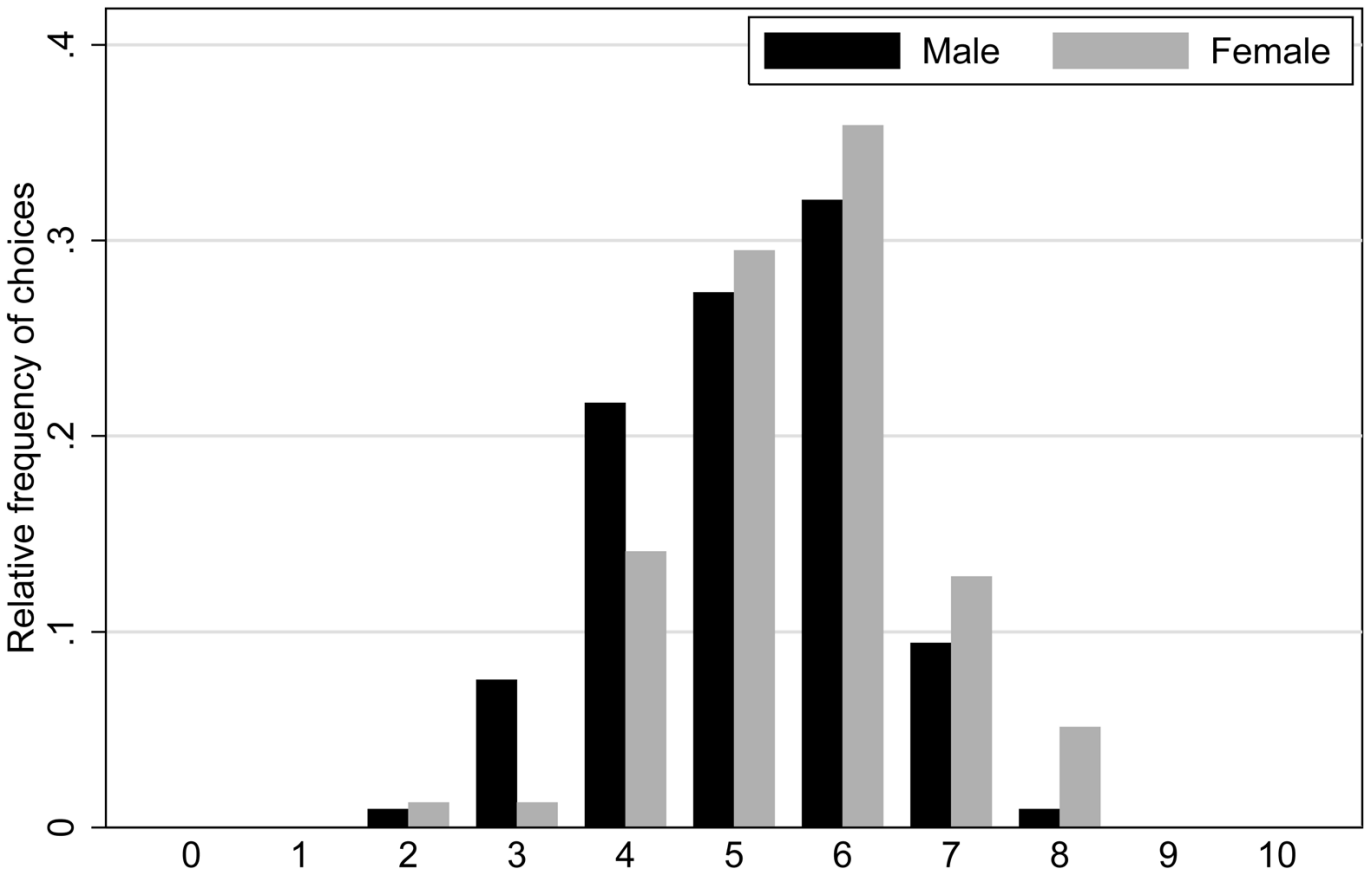
Risk preferences split by gender. The number of safe choices chosen by men (black) and women (grey).

We find that, on average, participants are risk averse. We also find that females are significantly more risk averse than men (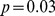). See Table 1 for summary statistics. Risk preferences are further illustrated in Figure 1, which contains a histogram of the number of safe choices, split by gender. Note that higher risk aversion in females is consistent with the literature (see e.g. [2], [21]).

**Table 1:** Summary statistics by gender.

	Males			Females			
	Mean	Std. Dev.	N	Mean	Std. Dev.	N	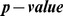
Age	21.12	3.43	125	21.10	2.17	94	0.48
Right-hand 2D:4D	0.94	0.03	125	0.96	0.03	94	0.00
Left-hand 2D:4D	0.94	0.03	125	0.96	0.03	94	0.00
Number of Safe Choices	5.14	1.18	106	5.56	1.16	78	0.03
Multiple switching	0.15	0.36	125	0.17	0.38	94	0.43

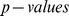
 are for one sided 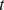-tests, except for the Number of Safe Choices, which is a Wilcoxon rank-sum test, and Multiple switching, which is a one sided Fisher's exact test.

**Figure 2:**
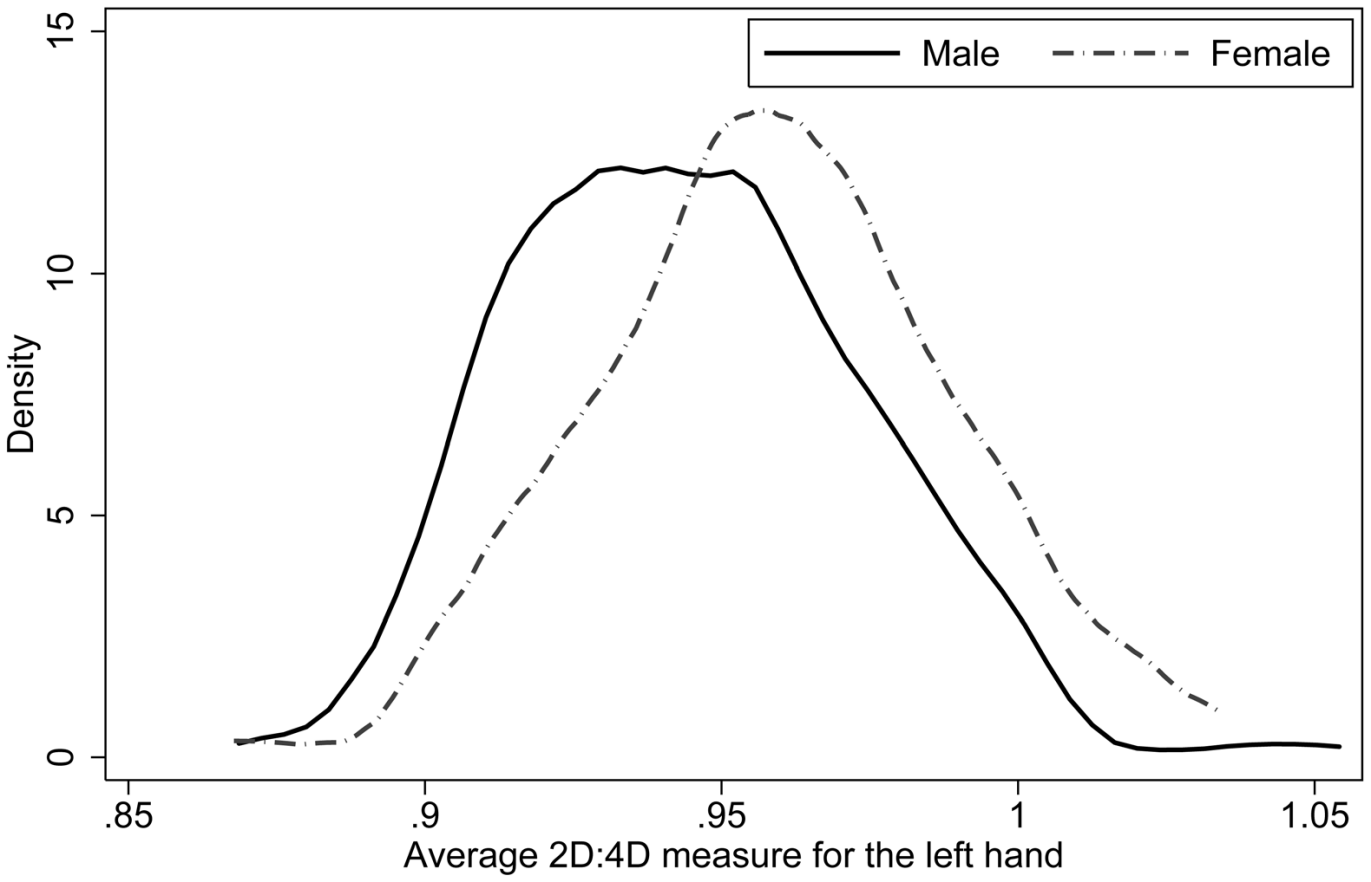
Kernel densities of left-hand 2D:4D. Kernel densities are estimated separately for men (solid) and women (dotted).

**Figure 3:**
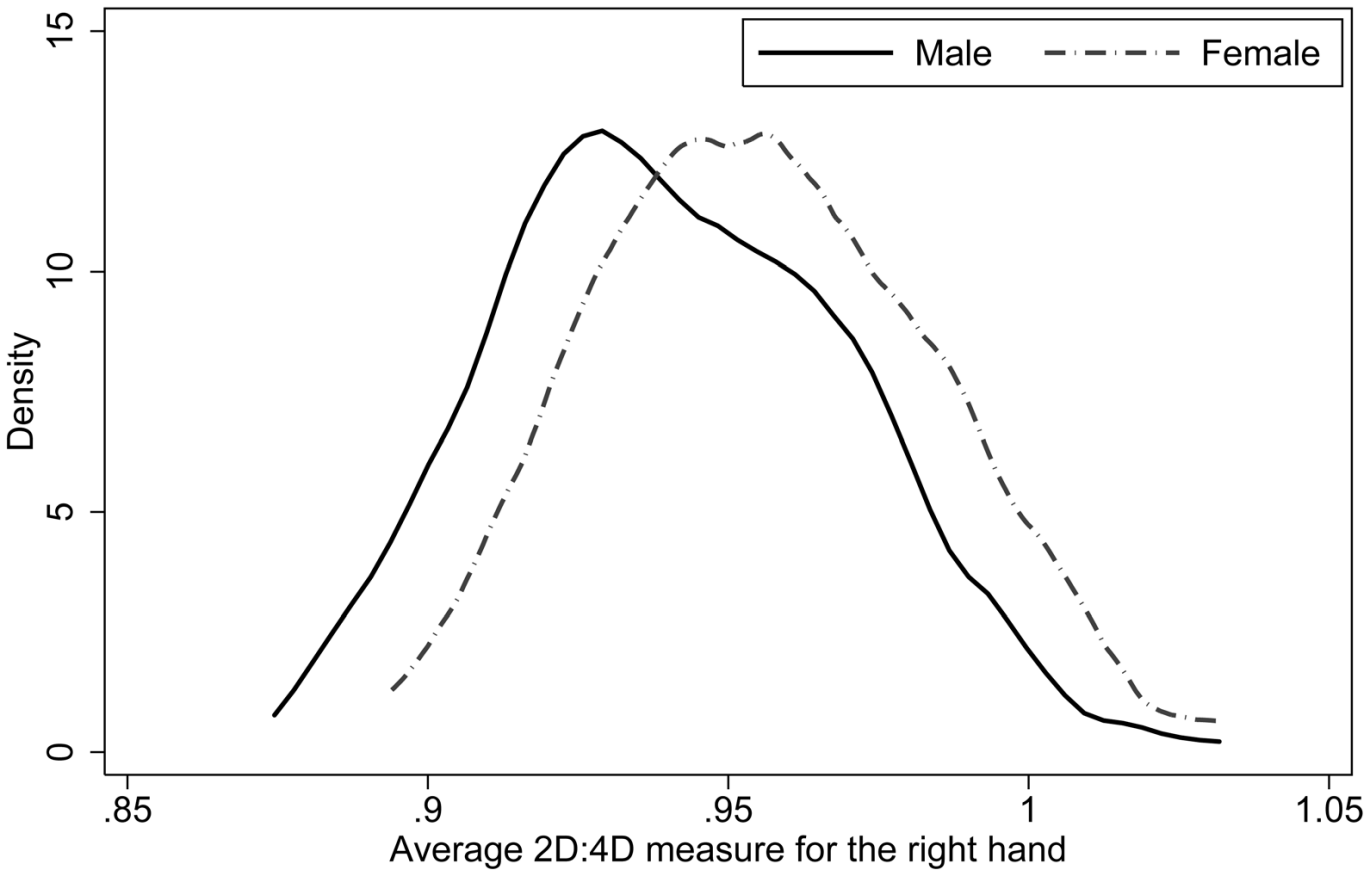
Kernel densities of right-hand 2D:4D. Kernel densities are estimated separately for men (solid) and women (dotted).

Our measure of 2D:4D is the average of five independent measures, by five research assistants. The average 2D:4D in our sample is 0.95 for both left and right hands. The average 2D:4D is 0.94 for both left and right male hands, and it is 0.96 for females. This gender difference is highly significant for both hands (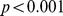). Table 1 contains the summary statistics and tests. Figures 2 and 3 contain kernel density plots of 2D:4D for both left and right hands respectively and are split by gender. Such sexual dimorphism in 2D:4D is typical (see e.g. [8]), and reflects higher (lower) prenatal exposure to testosterone (estrogen) among men.

This paper provides the first data of 2D:4D for Ladinos. Much of the existing literature either does not identify the racial composition of their sample (e.g. [17]), or restricts attention to Caucasians (e.g. [22]). As such, it is worthwhile to compare 2D:4D in Ladinos to 2D:4D in Caucasians. We compare our data against a large sample of Caucasians. A subset of this sample is analyzed in [22] (a detailed description of the entire Caucasian sample can be found in Table S1). Note that high resolution scans and repeated measures are utilized in both of our samples, which allows for a high level of comparability. A word of caution is appropriate with regards to this comparison exercise for two reasons. First, neither the Caucasian sample nor our sample of Ladinos is a truly random sample of their respective populations. Rather, participants are primarily college students. Second, there is some evidence that differences in 2D:4D exist between Caucasians from different countries [23]. Since the Caucasian sample we consider is from Spain, it is important to note that it is not a representative sample of all Caucasians. Nonetheless, we feel that comparison of these samples remains worthwhile.

We provide kernel density plots for both Caucasians and Ladinos. Figure S1 illustrates right-hand male 2D:4D ratios. Figures S2-4 contain the same for left-hand male, right-hand female and left-hand female respectively. The primary observation is that Caucasians have higher 2D:4D ratios than Ladinos, regardless of gender or hand (differences of mean t-tests: male, right hand 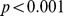; male, left hand 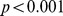; female, right hand 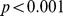; female, left hand 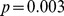). Further, the dispersion is smaller for Ladinos, again regardless of gender or hand. Table S1 contains a detailed comparison of these two samples.

### Regression analysis

To estimate the effect of 2D:4D ratio on risk preferences we estimate a series of ordered probits with robust standard errors where the (discrete) dependent variable is the number of safe choices a participant chose in the risk elicitation task. Standard errors are clustered at the experimental session level. Recall that we drop observations with inconsistent choices. We run several robustness checks. First, we estimate all models using ordered logits. Second, we estimate all models using the median 2D:4D measure, rather than the average. Third, we estimate all models using probits in which the dependent variable is equal to one if a participant is risk averse, and zero otherwise. Lastly, we estimate all models with the inconsistent observations, and use a dummy variable to control for inconsistency. As all results are robust to these changes, the estimates are not reported here.

We estimate separate models for left-hand and right-hand 2D:4D, since there is some evidence that right-hand 2D:4D is a better measure of prenatal exposure to sex hormones (see e.g. [8]), although this issue has not been investigated for Ladinos. Additionally, for each hand, we estimate separately for females and males as well as for the pooled data since some studies find that 2D:4D predicts risk preferences for males, but not females [14].

When considering the pooled data we estimate seven specifications for each hand. The first regression reported only includes gender as an explanatory variable, which we use as a baseline for model selection. The second specification only includes 2D:4D. In the third reported regression we add 2D:4D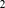. In the fourth specification we consider 2D:4D and gender. The fifth specification includes 2D:4D, 2D:4D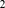 and gender. The sixth considers 2D:4D, gender and an interaction between the two. The seventh adds 2D:4D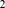 and an interaction between these additions. The final regression is the same as the seventh, except that we also control for age.

We first consider results for the right hand, which are contained in Table 2. The first important result is that neither 2D:4D nor 2D:4D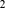 is significant in any regression in which it is included. Further, for any regression in which gender is excluded, we are unable to reject the joint insignificance of the regression using a Wald test. Finally, note that both the Bayesian Information Criterion (BIC) and the Akaike information criterion (AIC) favor the model which only includes gender. We therefore conclude that right-hand 2D:4D has no explanatory power for the risk preferences of Ladinos.

**Table 2:** Ordered probits on the number of safe choices for both males and females using right-hand 2D:4D.

	(1)	(2)	(3)	(4)	(5)	(6)	(7)	(8)
2D:4D		3.31	71.21	1.66	46.05	3.57	−41.70	−32.84
		(2.82)	(161.10)	(3.03)	(155.09)	(4.17)	(220.70)	(223.00)
2D:4D^2^			−35.89		−23.46		24.03	19.30
			(85.55)		(82.43)		(117.85)	(119.09)
Female	0.38**			0.35*	0.35*	5.13	−74.94	−63.29
	(0.14)			(0.15)	(0.14)	(5.65)	(151.91)	(154.07)
2D:4D  Female						−5.05	163.61	138.49
						(6.03)	(320.84)	(325.70)
2D:4D 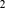  Female							−88.75	−75.23
							(169.35)	(172.06)
Age								0.03
								(0.03)
Pseudo R 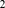	0.010	0.002	0.003	0.011	0.011	0.012	0.012	0.013
BIC	609.0281	613.4485	618.4648	613.9102	619.0409	618.4001	628.6028	633.4068
AIC	586.5236	590.9439	592.7453	588.1907	590.1064	589.4657	593.2385	594.8276
Observations	184	184	184	184	184	184	184	184
Number of clusters	27	27	27	27	27	27	27	27
Wald test statistic	7.638455	1.381971	2.000838	8.587886	8.592126	12.41138	13.26349	24.4814
Wald 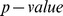	.0057137	.2397657	.3677254	.013651	.0352353	.006099	.0210308	.0004258

Robust standard errors, clustered at the session level, are in parentheses. 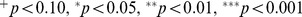

Results for the pooled data for the left hand are contained in Table 3. The reported specifications are the same as that of the right hand. Notice that once again both the BIC and AIC favor the model which only includes gender as an explanatory variable. Further, neither 2D:4D nor 2D:4D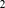 is significant at the 5% level in any regression in which it is included. Likewise, in a regression which does not include gender as an explanatory variable, we are unable to reject joint insignificance at the 5% level using Wald tests. Note that when we only include 2D:4D, it is significant at the 10% level. However, once we add gender, this is no longer the case. This indicates that the marginal significance of 2D:4D when it is the sole variable is driven by gender difference in 2D:4D. Once gender is held constant, 2D:4D is once again insignificant. Given this, we conclude that left-hand 2D:4D has no explanatory power for the risk preferences of Ladinos.

**Table 3:** Ordered probits on the number of safe choices for both males and females using left-hand 2D:4D.

	(1)	(2)	(3)	(4)	(5)	(6)	(7)	(8)
2D:4D		4.77+	56.67	3.44	53.25	3.45	234.60	255.26
		(2.67)	(114.13)	(2.59)	(115.90)	(3.37)	(197.31)	(193.93)
2D:4D 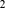			−27.28		−26.19		−121.73	−132.71
			(60.08)		(60.95)		(104.24)	(102.56)
Female	0.38**			0.32*	0.32*	0.37	196.77	215.81
	(0.14)			(0.13)	(0.13)	(5.37)	(134.28)	(133.86)
2D:4D  Female						−0.04	−413.43	−453.58
						(5.66)	(283.65)	(282.95)
2D:4D 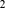  Female							217.32	238.45
							(149.71)	(149.44)
Age								0.04
								(0.03)
Pseudo R 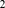	0.010	0.006	0.006	0.013	0.013	0.013	0.018	0.020
BIC	609.0281	611.4458	616.4753	612.5826	617.627	617.7975	625.2157	629.4786
AIC	586.5236	588.9412	590.7558	586.8631	588.6925	588.863	589.8514	590.8994
Observations	184	184	184	184	184	184	184	184
Number of clusters	27	27	27	27	27	27	27	27
Wald test statistic	7.638455	3.202329	3.563388	8.321071	8.87507	8.525426	11.76268	20.97335
Wald 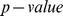	.0057137	.0735335	.1683527	.0155992	.0309988	.0363136	.0381884	.001855

Robust standard errors, clustered at the session level, are in parentheses.

+ *p*<0.10.

*p<0.05.

**p<0.01.

***p<0.001

Results using the male subsample are contained in Table 4. We estimate three models for each hand. In the first, we only include 2D:4D. In the second we also included 2D:4D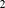. In the third we add age. In all relevant specifications, 2D:4D and 2D:4D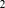 are insignificant. Further, in all specifications we are unable to reject joint insignificance of all variables (despite the fact that age is significant for both hands). Therefore, we conclude that 2D:4D does not explain risk preferences for Ladino males.

**Table 4:** Ordered probits on the number of safe choices for males.

	(Right 1)	(Right 2)	(Right 3)	(Left 1)	(Left 2)	(Left 3)
2D:4D	4.00	−56.97	−32.13	3.58	242.57	293.15
	(4.14)	(227.66)	(233.62)	(3.34)	(196.87)	(195.45)
2D:4D 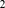		32.37	19.11		−125.86	−152.73
		(121.63)	(124.77)		(104.02)	(103.46)
Age			0.07^+^			0.09*
			(0.04)			(0.05)
Pseudo R 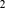	0.004	0.004	0.009	0.003	0.010	0.017
BIC	361.4567	366.0192	369.157	361.6122	364.1182	366.4087
AIC	342.8126	344.7117	345.1861	342.9681	342.8107	342.4377
Observations	106	106	106	106	106	106
Number of clusters	27	27	27	27	27	27
Wald test statistic	.9344658	.9010023	3.457829	1.143379	3.333867	5.392458
Wald 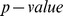	.3337052	.6373087	.3262728	.2849394	.1888253	.1452142

Robust standard errors, clustered at the session level, are in parentheses. 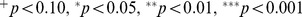

The corresponding ordered probits for the female subsample are contained in Table 5. The results mirror those of the male subsample, with the exception that age is not significant for either hand. Regardless of the specification or hand considered, no variable is individually significant, and we are unable to reject the null hypothesis that all variables are jointly insignificant using Wald tests. As such, we conclude that 2D:4D does not explain the risk preferences of Ladino females.

**Table 5:** Ordered probits on the number of safe choices for females.

	(Right 1)	(Right 2)	(Right 3)	(Left 1)	(Left 2)	(Left 3)
2D:4D	−1.84	108.83	117.39	3.08	−176.83	−176.53
	(4.11)	(265.89)	(270.34)	(4.19)	(194.54)	(196.29)
2D:4D 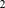		−58.04	−62.66		94.36	94.20
		(140.02)	(142.42)		(102.02)	(102.93)
Age			−0.01			−0.00
			(0.05)			(0.04)
Pseudo R 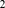	0.001	0.001	0.001	0.002	0.006	0.006
BIC	269.2578	273.4772	277.7748	268.8441	272.2544	276.611
AIC	252.7609	254.6235	256.5644	252.3471	253.4007	255.4006
Observations	78	78	78	78	78	78
Number of clusters	26	26	26	26	26	26
Wald test statistic	.2017513	.298814	.3711948	.5408419	1.368441	1.370417
Wald 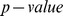	.6533109	.8612185	.9461265	.462084	.5044833	.7124836

Robust standard errors, clustered at the session level, are in parentheses. 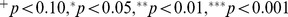

In summary, we find that the 2D:4D does not explain the risk preferences of Ladino males or females. Studies which consider racially homogenous Caucasian samples have found some evidence of a relationship [13], [14]. However, studies that have analyzed racially heterogeneous samples have typically not found such a relationship [15]–[17]. Our results demonstrate the importance of exploring the behavioral effects of 2D:4D in non-Caucasian racial groups, since positive results may not be robust to other racial groups.

## Methods

### Ethics statement

This project was approved by the Internal Review Board at Gettysburg College. All participants signed consent forms prior to participation. No deception was used.

### Experimental sessions

In each experimental session twelve participants complete a series of tasks via a computer interface that was programmed using z-Tree [24]. In this paper, we focus on the last task, which is designed to elicit risk preferences. All but one of the earlier tasks are designed to elicit preferences regarding competition using a modified version of the tasks used by [25]. These tasks involve adding up series of two digit numbers under either a piece rate payment scheme, a tournament payment scheme, or a mix of the two. The remaining task elicits beliefs regarding their relative performance in a task, in which they add numbers. Subjects do not receive any feedback that could affect their perceived wealth during the tasks. Note that we cannot rule out the possibility that the previous tasks affected behavior in the risk elicitation task. However, in the unlikely event that the previous tasks affected behavior in the risk elicitation task, there is no reason to expect that this effect is correlated with 2D:4D.

The risk elicitation task is similar to that of [19] and involves ten binary choices between two lotteries. Once all tasks are complete, one task is randomly and publically chosen for payment using a bingo cage. This is done for two reasons. First, this payment method is both incentive compatible and avoids portfolio or wealth effects. Second, the public use of a bingo cage ensures that the randomization is transparent to all participants, and facilitates easy understanding of the associated probabilities. Once participants are informed of their payoffs, they are asked to complete a short post-experimental survey. Once they complete this survey, they are called one by one to have their hands scanned using an Epson Perfection V30 scanner (for subsequent measurement of the 2D:4D ratio) and to receive their individual cash payment in private.

All sessions were run at Centro Vernon Smith de Economía Experimental at Universidad Francisco Marroquín. Subjects were predominantly undergraduates of Universidad Francisco Marroquín (UFM), although some subjects were students at surrounding universities. Each session lasted for approximately one and a half hours. Subjects were paid a 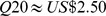 for showing-up, in addition to their earnings from the experiment. All monetary amounts in the experiment were denominated in Quetzales.

### Risk preferences elicitation

The risk elicitation task is very similar to that of [19]. Immediately prior to the risk elicitation task, participants are shown a short video containing the instructions. The text of the instructions (translated from the original Spanish) can be found in Text S1, and the video itself is Video S1. This video contains figures to illustrate the choices associated with the task, and contains an example to facilitate understanding.

There are ten decisions in this task. Each decision involves a choice between two lotteries: lottery A and lottery B. These decisions, framed as choices between option A and option B, are illustrated in Table 6. Each lottery has two possible payoffs, one higher than the other. In lottery A the high payoff is 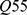 and the low payoff is 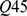. In lottery B they are 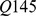 and 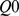 (these amounts are approximately US$5.75, US$7.05, US$18.60 and US$0, respectively). The probability (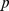) that the high payoff is chosen is the same for both lotteries in each of the ten decisions. In the first decision, 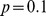, and it increases in each successive decision by 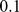 so that for decision ten 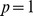. To ensure that 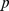 is easily understood by the participants, each lottery is presented as a choice of either a blue or green ball using a bingo cage. There are ten balls total, with 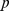 being the proportion of blue balls in the cage.

**Table 6:** Summary of risk eliciation task.

		Lottery A			Lottery B		
Choice	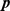	High amount	Low amount	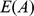	High amount	Low amount	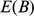
1	0.1	55	45	46	145	0	14.5
2	0.2	55	45	47	145	0	29
3	0.3	55	45	48	145	0	43.5
4	0.4	55	45	49	145	0	58
5	0.5	55	45	50	145	0	72.5
6	0.6	55	45	51	145	0	87
7	0.7	55	45	52	145	0	101.5
8	0.8	55	45	53	145	0	116
9	0.9	55	45	54	145	0	130.5
10	1	55	45	55	145	0	145

Notice that the range of payoffs in lottery A is narrow relative to that of lottery B. Also note that in the first decision, the probability of obtaining the higher amount is relatively low and therefore, the expected value of lottery A is greater than that of lottery B. As probability mass is shifted to the higher payoff, the expected value of lottery A increases less than that of lottery B. In decision four, and each subsequent decision, the expected value of lottery B is greater than that of lottery A. Thus, a risk neutral person choosing according to the relative expected utility of the two lotteries would choose lottery A in the first three decisions, and switch to lottery B in the fourth decision and all subsequent decisions. As such, the switch from lottery A to lottery B allows us to classify the participants according to their associated risk preferences. A risk seeking participant will switch before decision four, while a risk averse participant will switch after decision four.

It is, of course, possible that a participant switches between lottery A and lottery B multiple times. This could be because the participant is indifferent between the associated lotteries, or be due to decision errors or confusion. Either way, multiple switching involves inconsistent choices and makes it unclear how to classify risk preferences precisely. We opted to allow multiple switch points (this could be easily avoided by, for instance, simply asking to report the first decision for which they would prefer lottery B) because we wish to be able to control for this in our analysis.

In our analysis, we use the number of times a participant expresses a preference for lottery A as our main classification of risk aversion, and refer to this measure as the number of safe choices. This measure is the dependent variable in our econometric analysis. Note that for the reported regressions, we restricted attention to those participants who made consistent choices. All regression results are robust to using the full sample and when we control for individuals who switched multiple times, the coefficient is not statistically different from zero.

After participants make their choices, one of the experimental tasks is randomly chosen for payment. If the risk elicitation task is chosen, one of the ten decisions is randomly selected using a ten-sided die. Ten balls are then placed in the bingo cage. The number randomly selected by the ten-sided die corresponds to the decision number and determines the number of blue balls. One of these balls is publically drawn. If the chosen ball is blue, then each participant in the session receives the higher payment from the lottery chosen in that decision. If the chosen ball is green, each participant receives the lower payment.

### Digit ratio measurement

Each participant has her hands scanned, face down with fingers straight and spread apart, and with slight downward pressure. We ask participants to spread their fingers slightly because when asked to keep fingers together one tends to bend the hand slightly, elevating the base of the fingers, and making the basal creases difficult to discern. Any jewelry is removed. If there is a problem with the scan, the process is repeated. The use of a digital scan is a common method of measurement, and has been shown to be the most reliable measure commonly used [26]. An example scan can be seen in Figure S5.

The measurement of each finger is done from the center of the basal crease to the tip of the finger. All measurements are done using Autometric software, which is designed to measure 2D:4D [27].

The scans are randomly sorted into batches of approximately 20 images. Five research assistants independently measure both hands of each image in each batch. Each assistant measures each batch separately. The order in which the batches were given to each assistant is randomized so that if measurement error changes over time, this effect would be random. Splitting the measurement into batches also breaks it up into smaller tasks, to further reduce the effects of fatigue or boredom.

We also randomly inserted thirty-six images into two batches without informing the research assistants in order to evaluate intra-rater consistency. That is, thirty-six images were randomly chosen and added to a second batch, so that each assistant would measure each of these images twice.

The between-rater intraclass correlation coefficients are 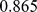 (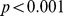) for the left hand and 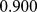 (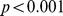) for the right hand, indicating a high degree of consistency. Further, statistical analysis of between-rater consistency can be found in Table S2. Within-rater measures also indicate a high degree of consistency. Intraclass correlation coefficients range between 0.830 and 0.961 for the left hand, and between 0.794 and 0.974 for the right hand. All within-rater intraclass correlation coefficients can be found in Table S3.

In our analysis, we use the average of the five measurements for both the right and left hand. This is consistent with the measure utilized in [22], [28]. Note that our analysis is robust to using alternative measures, such as the median.

## Supporting Information

Figure S1 **Kernel densities of right-hand male 2D:4D for Ladinos and Caucasians.** BEEGKN refers to the data utilized in [22]. Note that this is a subset of a larger data set. We compare against the full data set.
(TIF)

Figure S2 **Kernel densities of left-hand male 2D:4D for Ladinos and Caucasians.** BEEGKN refers to the data utilized in [22]. Note that this is a subset of a larger data set. We compare against the full data set.
(TIF)

Figure S3 **Kernel densities of right-hand female 2D:4D for Ladinos and Caucasians.** BEEGKN refers to the data utilized in [22]. Note that this is a subset of a larger data set. We compare against the full data set.
(TIF)

Figure S4 **Kernel densities of left-hand female 2D:4D for Ladinos and Caucasians.** BEEGKN refers to the data utilized in [22]. Note that this is a subset of a larger data set. We compare against the full data set.
(TIF)

Figure S5 **Example scan of hands.**
(TIF)

Table S1 **Comparison between our sample of Ladinos and the Caucasian sample in BEEGKN.** BEEGKN refers to the data utilized in [22]. Note that this is a subset of a larger data set. We compare against the full data set.
(DOCX)

Table S2 **Between-rater Spearman correlation coefficients for 2D:4D measures.**
(DOCX)

Table S3 **Within-rater consistency for 2D:4D measures.**
(DOCX)

Text S1 **Instructions for the risk elicitation task.** The text corresponds to a video which contains and is translated from the original Spanish.
(PDF)

Video S1 **This video contains the instructions for the risk elicitation task as it was shown to participants.**
(MP4)

## References

[1] Byrnes JP, Miller DC, Schafer WD (1999) Gender differences in risk taking: a meta-analysis. Psychological bulletin 125: 367.

[2] Eckel CC, Grossman PJ (2008) Differences in the economic decisions of men and women: experimental evidence. Handbook of experimental economics results 1: 509–519.

[3] Goy RW, McEwen BS (1980) Sexual differentiation of the brain. Cambridge, MA: Mit Press.

[4] Manning JT, Scutt D, Wilson J, Lewis-Jones DI (1998) The ratio of 2nd to 4th digit length: a predictor of sperm numbers and concentrations of testosterone, luteinizing hormone and oestrogen. Human reproduction 13: 3000–3004. 9853845 10.1093/humrep/13.11.3000

[5] Lutchmaya S, Baron-Cohen S, Raggatt P, Knickmeyer R, Manning J (2004) 2nd to 4th digit ratios, fetal testosterone and estradiol. Early human development 77: 23–28. 15113628 10.1016/j.earlhumdev.2003.12.002

[6] Manning J, Kilduff L, Trivers R (2013) Digit ratio (2d: 4d) in klinefelter's syndrome. Andrology 1: 94–99. 23258636 10.1111/j.2047-2927.2012.00013.x

[7] Zheng Z, Cohn MJ (2011) Developmental basis of sexually dimorphic digit ratios. Proceedings of the National Academy of Sciences 108: 16289–16294. 10.1073/pnas.1108312108 PMC3182741 21896736

[8] Manning JT (2002) Digit ratio: a pointer to fertility, behavior, and health. Rutgers University Press.

[9] Voracek M, Manning JT, Dressler SG (2007) Repeatability and interobserver error of digit ratio (2d: 4d) measurements made by experts. American Journal of Human Biology 19: 142–146. 17160984 10.1002/ajhb.20581

[10] Hönekopp J, Bartholdt L, Beier L, Liebert A (2007) Second to fourth digit length ratio (2d: 4d) and adult sex hormone levels: new data and a meta-analytic review. Psychoneuroendocrinology 32: 313–321. 17400395 10.1016/j.psyneuen.2007.01.007

[11] Manning J, Henzi P, Venkatramana P, Martin S, Singh D (2003) Second to fourth digit ratio: ethnic differences and family size in english, indian and south african populations. Annals of human biology 30: 579–588. 12959899 10.1080/0301446032000112689

[12] Manning JT, Stewart A, Bundred PE, Trivers RL (2004) Sex and ethnic differences in 2nd to 4th digit ratio of children. Early Human Development 80: 161–168. 15500996 10.1016/j.earlhumdev.2004.06.004

[13] Garbarino E, Slonim R, Sydnor J (2011) Digit ratios (2d: 4d) as predictors of risky decision making for both sexes. Journal of Risk and Uncertainty 42: 1–26.

[14] Brañas-Garza P, Rustichini A (2011) Organizing effects of testosterone and economic behavior: Not just risk taking. PloS one 6: e29842. 22242144 10.1371/journal.pone.0029842 PMC3248440

[15] Apicella CL, Dreber A, Campbell B, Gray PB, Hoffman M, et al (2008) Testosterone and financial risk preferences. Evolution and Human Behavior 29: 384–390.

[16] Schipper B (2012) Sex hormones and choice under risk.

[17] Sapienza P, Zingales L, Maestripieri D (2009) Gender differences in financial risk aversion and career choices are affected by testosterone. Proceedings of the National Academy of Sciences 106: 15268–15273. 10.1073/pnas.0907352106 PMC2741240 19706398

[18] Instituto Nacional de Estadstica Guatemala (2011). Encuesta nacional de condiciones de vida. Available: http://www.ine.gob.gt/index.php/encuestas-de-hogares-y-personas/condiciones-de-vida.

[19] Holt C, Laury S (2002) Risk aversion and incentive effects. American economic review 92: 1644–1655.

[20] Eckel CC, Grossman PJ (2008) Forecasting risk attitudes: An experimental study using actual and forecast gamble choices. Journal of Economic Behavior & Organization 68: 1–17.

[21] Croson R, Gneezy U (2009) Gender differences in preferences. Journal of Economic Literature: 448–474.

[22] Bosch-Domènech A, Brañas-Garza P, Espín AM (2014) Can exposure to prenatal sex hormones (2d: 4d) predict cognitive reection? Psychoneuroendocrinology. 10.1016/j.psyneuen.2014.01.023 24703165

[23] Manning JT, Barley L, Walton J, Lewis-Jones D, Trivers R, et al (2000) The 2nd: 4th digit ratio, sexual dimorphism, population differences, and reproductive success: evidence for sexually antagonistic genes? Evolution and Human Behavior 21: 163–183. 10828555 10.1016/s1090-5138(00)00029-5

[24] Fischbacher U (2007) z-tree: Zurich toolbox for ready-made economic experiments. Experimental Economics 10: 171–178.

[25] Niederle M, Vesterlund L (2007) Do women shy away from competition? do men compete too much? Quarterly Journal of Economics 122: 1067–1101.

[26] Kemper CJ, Schwerdtfeger A (2009) Comparing indirect methods of digit ratio (2d: 4d) measurement. American Journal of Human Biology 21: 188–191. 18988284 10.1002/ajhb.20843

[27] DeBruine L (2006) Autometric software for measurement of 2d:4d ratios. Available: http://facelab.org/debruine/Programs/autometric.php.

[28] Brañas-Garza P, Kovářík J, Neyse L (2013) Second-to-fourth digit ratio has a non-monotonic impact on altruism. PloS one 8: e60419. 23593214 10.1371/journal.pone.0060419 PMC3622687

