# Supplementary figures and images for: Risk Preferences and Prenatal Exposure to Sex Hormones for Ladinos
Source: PLoS One. 2014 Aug 1;9(8):e103332. doi: 10.1371/journal.pone.0103332 (PMC4118870; doi:10.1371/journal.pone.0103332)

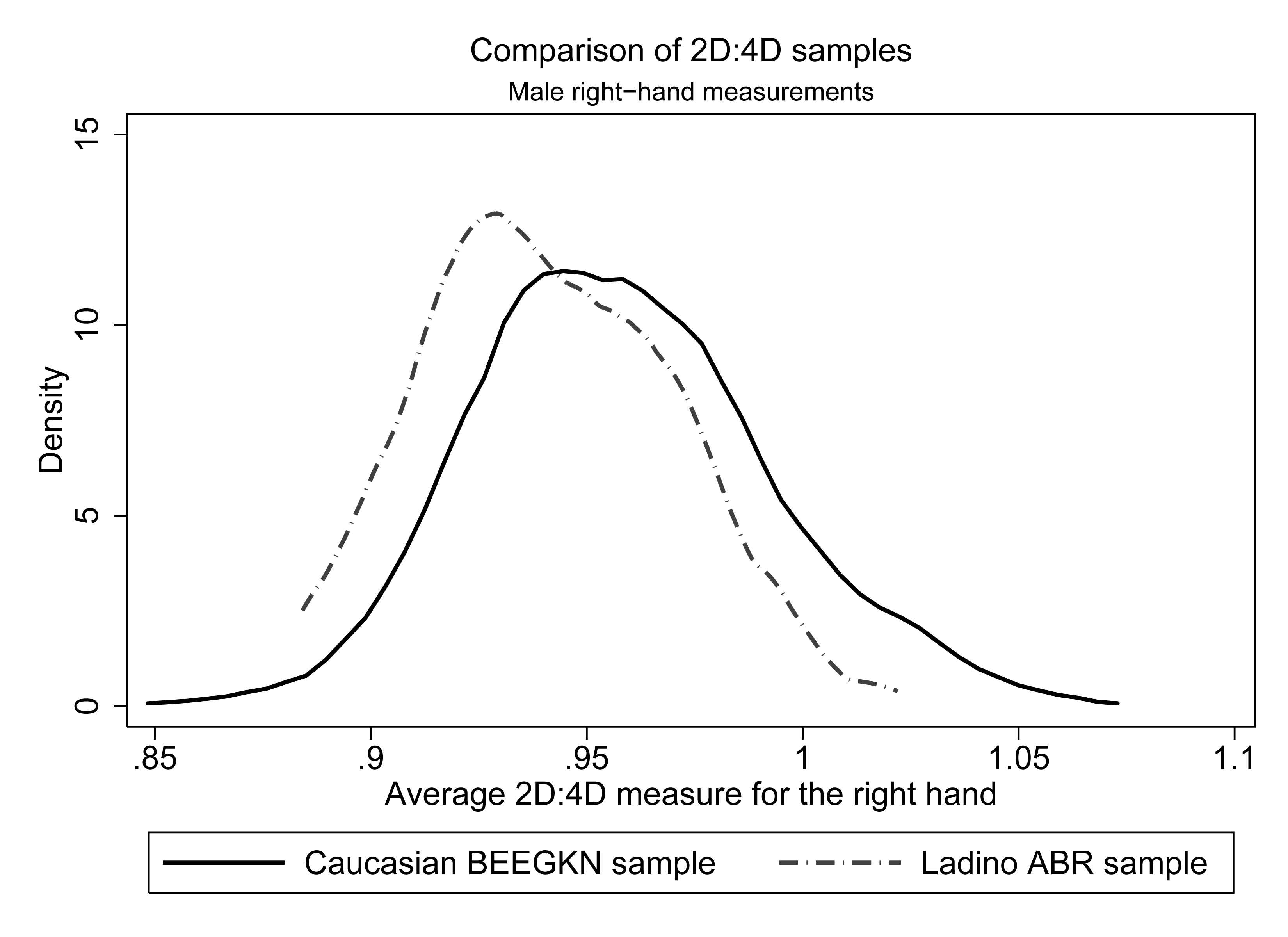

Supplement: Figure S1 — Kernel densities of right-hand male 2D:4D for Ladinos and Caucasians. BEEGKN refers to the data utilized in [22]. Note that this is a subset of a larger data set. We compare against the full data set. (TIF) [file pone.0103332.s001.tif]

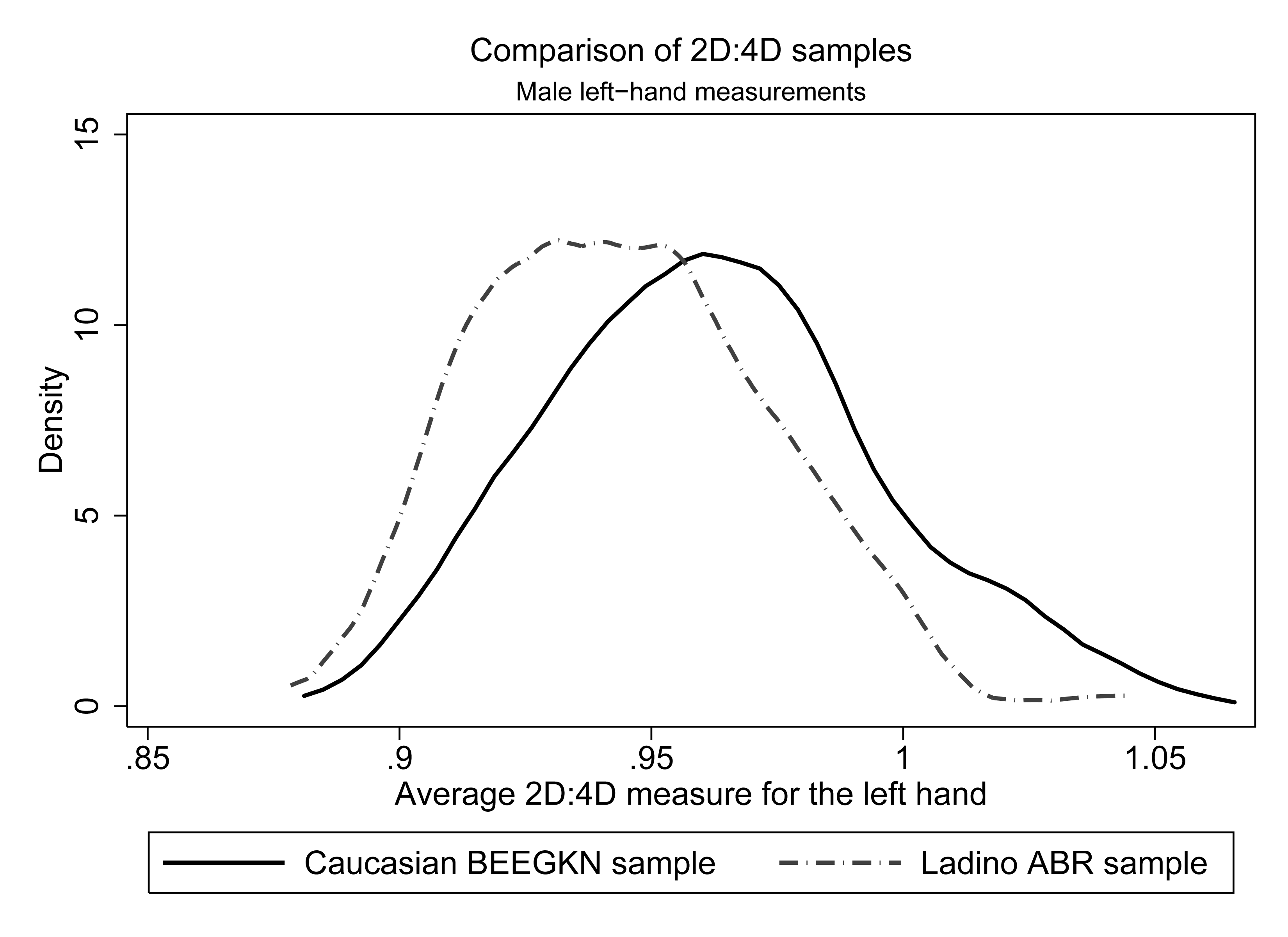

Supplement: Figure S2 — Kernel densities of left-hand male 2D:4D for Ladinos and Caucasians. BEEGKN refers to the data utilized in [22]. Note that this is a subset of a larger data set. We compare against the full data set. (TIF) [file pone.0103332.s002.tif]

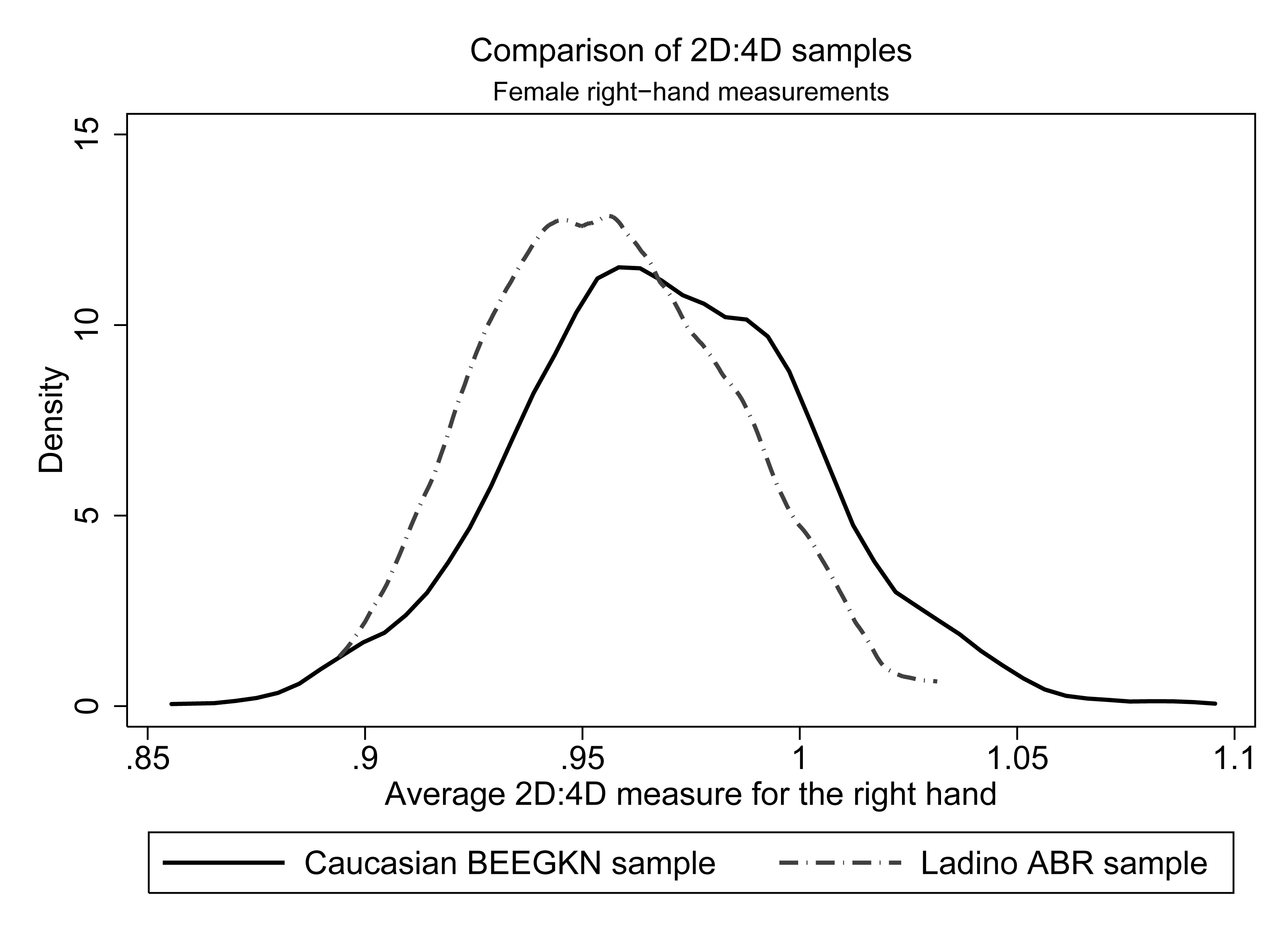

Supplement: Figure S3 — Kernel densities of right-hand female 2D:4D for Ladinos and Caucasians. BEEGKN refers to the data utilized in [22]. Note that this is a subset of a larger data set. We compare against the full data set. (TIF) [file pone.0103332.s003.tif]

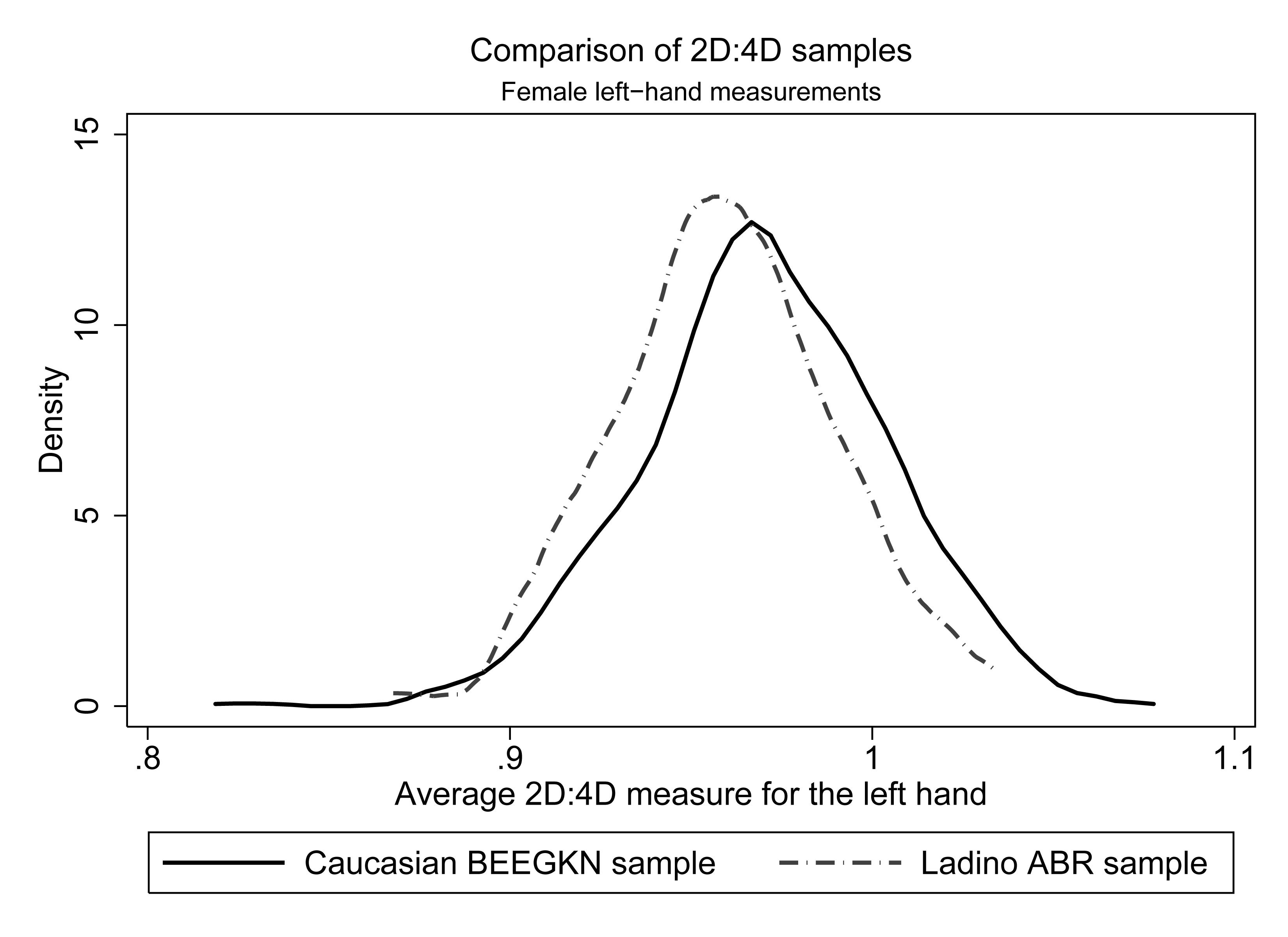

Supplement: Figure S4 — Kernel densities of left-hand female 2D:4D for Ladinos and Caucasians. BEEGKN refers to the data utilized in [22]. Note that this is a subset of a larger data set. We compare against the full data set. (TIF) [file pone.0103332.s004.tif]

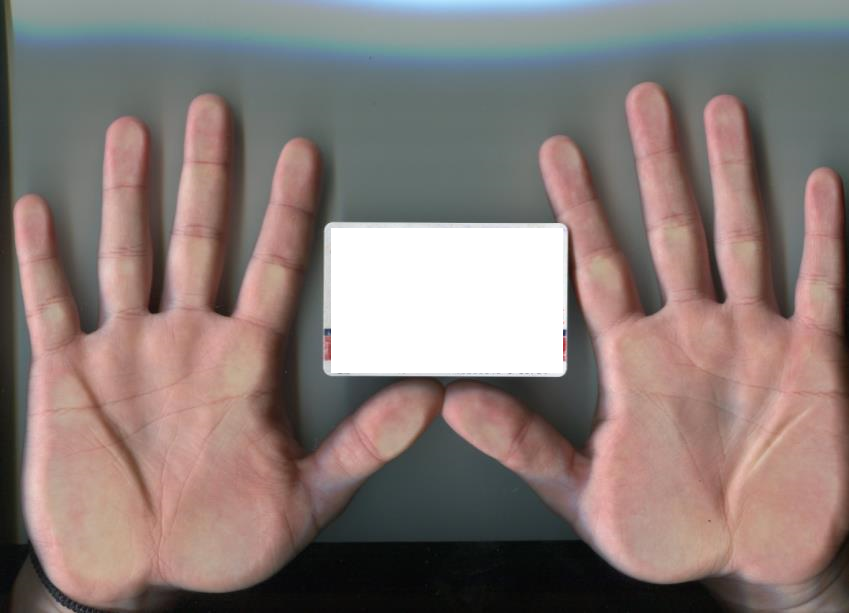

Supplement: Figure S5 — Example scan of hands. (TIF) [file pone.0103332.s005.tif]
