# Supplementary material for: Risk Preferences and Prenatal Exposure to Sex Hormones for Ladinos
Source: PLoS One. 2014 Aug 1;9(8):e103332. doi: 10.1371/journal.pone.0103332 (PMC4118870; doi:10.1371/journal.pone.0103332)
Supplement: Table S1 — Comparison between our sample of Ladinos and the Caucasian sample in BEEGKN. BEEGKN refers to the data utilized in [22]. Note that this is a subset of a larger data set. We compare against the full data set. (DOCX) [file pone.0103332.s006.docx]

**Table S1:** Comparison between our sample of Ladinos and the Caucasian sample in BEEGKN.

|  | **Caucasians** | | | |  | **Ladinos** | | | |
| --- | --- | --- | --- | --- | --- | --- | --- | --- | --- |
|  | Males | | Females | |  | Males | | Females | |
|  | *Right* | *Left* | *Right* | *Left* |  | *Right* | *Left* | *Right* | *Left* |
| **Min** | 0.8569 | 0.8897 | 0.8640 | 0.8269 |  | 0.8842 | 0.8784 | 0.8940 | 0.8678 |
| **1%** | 0.8848 | 0.8969 | 0.8952 | 0.8869 |  | 0.8848 | 0.8876 | 0.8940 | 0.8678 |
| **5%** | 0.9062 | 0.9115 | 0.9138 | 0.9148 |  | 0.8972 | 0.9038 | 0.9110 | 0.9112 |
| **10%** | 0.9176 | 0.9214 | 0.9268 | 0.9260 |  | 0.9034 | 0.9116 | 0.9208 | 0.9180 |
| **25%** | 0.9342 | 0.9396 | 0.9470 | 0.9497 |  | 0.9204 | 0.9226 | 0.9370 | 0.9414 |
| **50%** | 0.9555 | 0.9612 | 0.9674 | 0.9698 |  | 0.9366 | 0.9434 | 0.9541 | 0.9591 |
| **75%** | 0.9775 | 0.9830 | 0.9922 | 0.9934 |  | 0.9620 | 0.9630 | 0.9744 | 0.9806 |
| **90%** | 1.0008 | 1.0084 | 1.0106 | 1.0141 |  | 0.9810 | 0.9854 | 0.9954 | 0.9960 |
| **95%** | 1.0194 | 1.0217 | 1.0261 | 1.0248 |  | 0.9880 | 0.9922 | 0.9994 | 1.0114 |
| **99%** | 1.0404 | 1.0409 | 1.0480 | 1.0466 |  | 1.0050 | 1.0076 | 1.0316 | 1.0340 |
| **Max** | 1.0643 | 1.0572 | 1.0871 | 1.0695 |  | 1.0220 | 1.0444 | 1.0316 | 1.0340 |
|  |  |  |  |  |  |  |  |  |  |
| **Mean** | 0.9578 | 0.9625 | 0.9691 | 0.9702 |  | 0.9407 | 0.9445 | 0.9557 | 0.9589 |
| **Std. Dev.** | 0.0337 | 0.0326 | 0.0336 | 0.0333 |  | 0.0284 | 0.0285 | 0.0278 | 0.0300 |
|  |  |  |  |  |  |  |  |  |  |
| **Skewness** | 0.2818 | 0.2987 | 0.1004 | -0.1650 |  | 0.2352 | 0.4042 | 0.2334 | -0.0401 |
| **p-value** | 0.0164 | 0.0111 | 0.3210 | 0.1044 |  | 0.2639 | 0.0600 | 0.3290 | 0.8654 |
|  |  |  |  |  |  |  |  |  |  |
| **Kurtosis** | 3.0621 | 2.7686 | 3.1284 | 3.3068 |  | 2.5735 | 3.1368 | 2.6622 | 3.1616 |
| **p-value** | 0.6655 | 0.3332 | 0.4527 | 0.1381 |  | 0.3241 | 0.5431 | 0.6059 | 0.5124 |
|  |  |  |  |  |  |  |  |  |  |
| **Observations** | 437 | 437 | 574 | 574 |  | 125 | 125 | 94 | 94 |

Note: BEEGKN refers to the data utilized in Bosch-Domènech *et al.* (2014). Note that data used in Bosch-Domènech *et al.* (2014) is a subset of a larger data set. We compare against the full data set. A detailed description of the full data set can be found at https://sites.google.com/site/pablobranasgarza/2d-4d/a-large-sample.
