# Supplementary material for: Risk Preferences and Prenatal Exposure to Sex Hormones for Ladinos
Source: PLoS One. 2014 Aug 1;9(8):e103332. doi: 10.1371/journal.pone.0103332 (PMC4118870; doi:10.1371/journal.pone.0103332)
Supplement: Table S2 — Between-rater Spearman correlation coefficients for 2D:4D measures. (DOCX) [file pone.0103332.s007.docx]

**Table S2:** Between-rater Spearman correlation coefficients for 2D:4D measures.

|  | **Left-hand Measures** | | | | |
| --- | --- | --- | --- | --- | --- |
|  | Assistant 1 | Assistant 2 | Assistant 3 | Assistant 4 | Assistant 5 |
| Assistant 1 | 1 |  |  |  |  |
| Assistant 2 | 0.9373* | 1 |  |  |  |
| Assistant 3 | 0.8977* | 0.8810* | 1 |  |  |
| Assistant 4 | 0.8907* | 0.8740* | 0.8381* | 1 |  |
| Assistant 5 | 0.9330* | 0.9186* | 0.8744* | 0.8712* | 1 |
|  |  |  |  |  |  |
|  | **Right-hand Measures** | | | | |
|  | Assistant 1 | Assistant 2 | Assistant 3 | Assistant 4 | Assistant 5 |
| Assistant 1 | 1 |  |  |  |  |
| Assistant 2 | 0.9373* | 1 |  |  |  |
| Assistant 3 | 0.8753* | 0.9048* | 1 |  |  |
| Assistant 4 | 0.9175* | 0.9155* | 0.8777* | 1 |  |
| Assistant 5 | 0.9267* | 0.9252* | 0.8883* | 0.9303* | 1 |
