# Supplementary material for: Risk Preferences and Prenatal Exposure to Sex Hormones for Ladinos
Source: PLoS One. 2014 Aug 1;9(8):e103332. doi: 10.1371/journal.pone.0103332 (PMC4118870; doi:10.1371/journal.pone.0103332)
Supplement: Table S3 — Within-rater consistency for 2D:4D measures. (DOCX) [file pone.0103332.s008.docx]

**Table S3:** Within-rater consistency for 2D:4D measures.

|  | **Left Hand Repeated Measures** | |
| --- | --- | --- |
|  | Intraclass Correlation | Standard Error |
| Assistant 1 | 0.9611 | 0.0128 |
| Assistant 2 | 0.9135 | 0.0278 |
| Assistant 3 | 0.9173 | 0.0266 |
| Assistant 4 | 0.8296 | 0.0523 |
| Assistant 5 | 0.8310 | 0.0519 |
|  |  |  |
|  | **Right Hand Repeated Measures** | |
|  | Intraclass Correlation | Standard Error |
| Assistant 1 | 0.9737 | 0.0087 |
| Assistant 2 | 0.9447 | 0.0181 |
| Assistant 3 | 0.7940 | 0.0620 |
| Assistant 4 | 0.8699 | 0.0408 |
| Assistant 5 | 0.9014 | 0.0315 |
