# Supplementary material for: Risk Preferences and Prenatal Exposure to Sex Hormones for Ladinos
Source: PLoS One. 2014 Aug 1;9(8):e103332. doi: 10.1371/journal.pone.0103332 (PMC4118870; doi:10.1371/journal.pone.0103332)
Supplement: Text S1 — Instructions for the risk elicitation task. The text corresponds to a video which contains and is translated from the original Spanish. (PDF) [file pone.0103332.s009.pdf]

This file contains the instructions, translated from the original Spanish, which were used for the risk elicitation task. This text corresponds to a video which contains the following decision sheet.

OPCIÓN A										Fila de Decisión	OPCIÓN B									
55	45	45	45	45	45	45	45	45	45	1	145	0	0	0	0	0	0	0	0	0
55	55	45	45	45	45	45	45	45	45	2	145	145	0	0	0	0	0	0	0	0
55	55	55	45	45	45	45	45	45	45	3	145	145	145	0	0	0	0	0	0	0
55	55	55	55	45	45	45	45	45	45	4	145	145	145	145	0	0	0	0	0	0
55	55	55	55	55	45	45	45	45	45	5	145	145	145	145	145	0	0	0	0	0
55	55	55	55	55	55	45	45	45	45	6	145	145	145	145	145	145	0	0	0	0
55	55	55	55	55	55	55	45	45	45	7	145	145	145	145	145	145	145	0	0	0
55	55	55	55	55	55	55	55	45	45	8	145	145	145	145	145	145	145	145	0	0
55	55	55	55	55	55	55	55	55	45	9	145	145	145	145	145	145	145	145	145	0
55	55	55	55	55	55	55	55	55	55	10	145	145	145	145	145	145	145	145	145	145

When the text refers to this decision sheet, it was shown on the screen. This video is available in Supplementary Video S1.

The instructions follow:

In this Activity you will not have to add more numbers. This time you will have two options – OPTION A and OPTION B – that will pay money depending on the color of the ball – green or blue – that is chosen by a bingo cage.

This table is then shown and explained.

If the ball is color…	OPTION A	OPTION B
…BLUE	Q55	Q145
…GREEN	Q45	Q0

You will see ten decision rows. The number of blue (and green) balls varies by decision row. For each row you must choose between OPTION A or OPTION B.
In the first decision row there is 1 blue ball (and 9 green balls); in the second decision row, there are 2 blue balls and 8 green ones…and so on until the tenth decision row 10, in which

there are 10 blue balls and 0 green ones. (Note that there will always be 10 balls; what changes is the mix of blue and green balls according to the decision row).

If this Activity is randomly selected for payment, we will throw a 10-sided die to choose a decision row and then we will draw a ball from the bingo cage to determine your payment, depending on the OPTION that you chose for that row.

Please observe Decision Row 1 (1 blue ball and 9 green balls).

OPTION A pays Q55 if the blue ball comes out; or pays Q45 if one (out of 9) of the green balls comes out.

OPTION B pays Q145 if the blue ball comes out; or pays Q0 if one (out of 9) of the green balls comes out.

Note that the probability of drawing a blue ball in this row is 10% (1 blue ball out of 10); the probability of drawing a green ball in this row is 90% (9 green balls out of 10).

As you move down the decision sheet the number of blue balls increases (and the number of green balls decreases). That is, the probability that the payment is defined by the blue ball (the higher payment) increases for both options.

In fact, for the tenth decision row (the last row), there are only blue balls for both options. Therefore, the payment will, with certainty, be the one corresponding to a blue ball (100% probability). That is, for this row, you must choose between Q55 (OPTION A) or Q145 (OPTION B).

If Activity 6 is randomly chosen for payment, your earnings will be determined as follows: First we will roll a ten-sided die to choose the decision row that will determine your earnings. (Of course, each decision row has the same probability of being chosen).
Then, we will put the number of blue and green balls which correspond with the chosen decision row into the bingo cage. We will draw a ball from the bingo cage and your earnings will be determined by the color of the chosen ball and the option that you selected in the decision row.

Here is an example. Suppose that your earnings are determined according to this Activity, and that the number rolled by the die is 7. This means that row 7 will determine your earnings and we will put 7 blue balls and 3 green balls in the bingo cage.

If the bingo cage selects a blue ball and for row 7 you chose OPTION A, your earnings would be Q55. If for decision row 7 you chose OPTION B, your earning would be Q145.

If the bingo cage selects a green ball and for row 7 you chose OPTION A, your earnings would be Q45. If for row 7 you chose OPTION B, your earning would be Q0.
In the next screen you will see 10 decision rows and you will have to select the option you prefer for each row.

If you have any questions before beginning, please raise your hand.
